# The Impact of Perceptual Adaptation and Real Exposure to Catastrophic Events on Facial Emotion Categorization

**DOI:** 10.3390/brainsci16010005

**Published:** 2025-12-19

**Authors:** Pasquale La Malva, Valentina Sforza, Eleonora D’Intino, Irene Ceccato, Adolfo Di Crosta, Rocco Palumbo, Alberto Di Domenico, Giulia Prete

**Affiliations:** Department of Psychology, “G. d’Annunzio” University of Chieti-Pescara, 66013 Chieti, Italy; pasquale.lamalva@unich.it (P.L.M.); valentina.sforza@unich.it (V.S.); eleonora.dintino@unich.it (E.D.); irene.ceccato@unich.it (I.C.); adolfo.dicrosta@unich.it (A.D.C.); rocco.palumbo@unich.it (R.P.); alberto.didomenico@unich.it (A.D.D.)

**Keywords:** facial emotion recognition, perceptual adaptation, contextual modulation, disaster exposure, pre-traumatic stress (Pre-Cl)

## Abstract

**Background/Objectives:** Facial expressions are central to nonverbal communication and social cognition, and their recognition is shaped not only by facial features but also by contextual cues and prior experience. In high-threat contexts, rapid and accurate decoding of others’ emotions is adaptively advantageous. Grounded in neurocognitive models of face processing and vigilance, we tested whether brief perceptual adaptation to emotionally salient scenes, real-world disaster exposure, and pre-traumatic stress reactions enhance facial-emotion categorization. **Methods:** Fifty healthy adults reported prior direct exposure to catastrophic events (present/absent) and completed the Pre-Traumatic Stress Reactions Checklist (Pre-Cl; low/high). In a computerized task, participants viewed a single adaptor image for 5 s—negative (disaster), positive (pleasant environment), or neutral (phase-scrambled)—and then categorized a target face as emotional (fearful, angry, happy) or neutral as quickly and accurately as possible. Performance was compared across adaptation conditions and target emotions and examined as a function of disaster exposure and Pre-Cl. **Results:** Emotional adaptation (negative or positive) yielded better performance than neutral adaptation. Higher-order interactions among adaptation condition, target emotion, disaster exposure, and Pre-Cl indicated that the magnitude of facilitation varied across specific facial emotions and was modulated by both experiential (exposed vs. non-exposed) and dispositional (low vs. high Pre-Cl) factors. These effects support a combined influence of short-term contextual tuning and longer-term experience on facial-emotion categorization. **Conclusions:** Brief exposure to emotionally salient scenes facilitates subsequent categorization of facial emotions relative to neutral baselines, and this benefit is differentially shaped by prior disaster exposure and pre-traumatic stress. The findings provide behavioral evidence that short-term perceptual adaptation and longer-term experiential predispositions jointly modulate a fundamental communicative behavior, consistent with neurocognitive accounts in which context-sensitive visual pathways and salience systems dynamically adjust to support adaptive responding under threat.

## 1. Introduction

Facial expressions are fundamental to human nonverbal communication and play a crucial role in social cognition. Accurately recognizing others’ facial emotions helps infer their mental and emotional states and guides adaptive behavior in everyday interactions [1,2]. Importantly, emotion recognition is not driven by facial features alone: it is also strongly shaped by the context and situation in which an expression occurs [3,4]. Extensive evidence shows that congruent environmental or bodily cues can disambiguate a facial expression, improving both the speed and accuracy of categorization [3]. In many cases, situational context or whole-body cues can even outweigh facial features when judging someone’s emotion [4,5]. Observers also tend to encode contextual details when labeling facial emotions. A face seen in a fitting context (e.g., fear in a dangerous scene, happiness in a celebratory scene) is recognized more readily, whereas the same face in an incongruent or ambiguous context is more likely to be misinterpreted [3,4,5].

From a neuroscientific perspective, these contextual influences are implemented by partly distinct face-processing streams. Brain regions that support relatively invariant identity information (e.g., fusiform gyrus) are dissociable from regions specialized for changeable aspects such as gaze and expression (e.g., superior temporal sulcus, STS), and from limbic structures (including amygdala and insula) that register emotional salience [6]. Repetition of the same identity reduces fusiform activity, whereas repeating the same expression across identities adapts STS. In parallel, the amygdala typically shows habituation to repeated emotional cues—an efficiency mechanism thought to prevent saturation while preserving sensitivity to novelty [7,8,9,10]. Together, these findings suggest that neural responses to faces and emotions can be recalibrated by recent experience, offering a possible mechanistic account for how context can “tune” perception.

Context sensitivity is especially consequential in emergencies. Rapid and accurate identification of others’ facial emotions provides real-time information about potential threats and others’ intentions. Under acute threat (e.g., during natural hazards), quickly detecting fear or alarm in nearby faces can prompt timely defensive or cooperative responses. From an evolutionary perspective, readiness to decode fear, anger, or relief is advantageous because it supports appropriate fight/flight/help behaviors [11]. Consistent with this, emotional cues—especially threat-related expressions—capture attention more readily than neutral stimuli, even when they are brief or presented in peripheral vision [11].

A growing body of literature indicates that extreme experiences can durably modulate sensitivity to facial emotions. Direct traumatic exposure appears to foster a form of “emotional expertise”, with survivors often outperforming non-exposed individuals on facial-expression recognition tasks [12,13]. For example, one cohort of earthquake survivors showed higher accuracy and faster responses across multiple emotions than unexposed controls, interpreted as increased emotional vigilance after trauma [12]. In another sample of earthquake-exposed individuals (including those without post-traumatic stress disorder, PTSD), accuracy was higher, particularly for negative expressions (fear/anger), compared with unexposed individuals, suggesting hypersensitivity to threat-signaling faces in a persistently alarming environment [13]. At the neural level, earthquake witnesses exhibited altered relationships between facial-emotion accuracy and large-scale network connectivity, with performance differentially coupled with visual cortices and default-mode hubs [14]. Converging structural and functional findings in disaster-exposed samples (e.g., thalamic alterations and attentional avoidance biases) point to experience-dependent retuning of circuits that monitor emotion and filter salient cues [15,16].

Perceptual adaptation provides an experimental lens on how recent exposure shapes subsequent perception. Sustained or repetitive stimulation temporarily shifts sensitivity, producing measurable aftereffects on later stimuli. Such adaptation is well documented for basic visual features and also for complex categories such as faces [3]. For facial expressions, exposure to pronounced smiles or frowns shifts the perceived neutrality of subsequent faces (contrastive aftereffects), indicating dynamic recalibration of what counts as “neutral” [3]. A similar contrastive aftereffect has also been shown for subliminal emotional expressions, even when adaptation stimuli were presented for only 5 s [17]. Crucially, these are high-level effects: expression adaptation arises beyond local feature fatigue, and repetition specific to identity versus expression engages different cortical loci (fusiform vs. STS) [6]. Limbic systems also show emotion-dependent repetition effects (amygdala habituation), supporting efficient allocation of processing resources [8,9,10]. Adaptation paradigms can thus be used to test whether extremely negative images transiently “tune” the system to favor subsequent emotion decoding.

Indirect (vicarious) exposure via media can heighten stress and anxiety even far from the event, showing that vividly viewing catastrophic scenes can elicit robust stress responses [18,19]. Two competing accounts are plausible. Repeated negative exposure may lead to desensitization (blunted reactivity and higher thresholds for detecting affect) or to sensitization/heightened vigilance (lower thresholds and faster detection). Desensitization is supported by links between habitual violent-media exposure and reduced emotional/physiological responses and empathy, whereas sensitization aligns with findings that salient affective primes can speed emotion detection under arousal [11,20,21,22].

Building on these considerations, we use a cross-categorical adaptation design (adapting to environmental scenes, testing on faces) to probe short-term contextual tuning and its interaction with longer-term experiential factors. Prior work shows that adapting to complex emotional scenes can bias subsequent evaluations, and that phase-scrambled versions of the same images preserve low-level properties while removing semantic content, providing a neutral adaptation baseline [23,24]. After brief (5 s) adaptation [17] to negative (disaster), positive (relaxing), or neutral (scrambled) scenes, participants rapidly categorized target faces (fearful, angry, happy, neutral) as either emotional or neutral. Furthermore, we considered real-world exposure (direct experience of catastrophic events) and trait predispositions indexed by the Pre-Traumatic Stress Reactions Checklist (Pre-Cl) [25]. We specifically contrasted two broad accounts: a desensitization view, according to which repeated exposure to negative material blunts responsiveness and raises detection thresholds, and a sensitization/heightened vigilance view, according to which emotionally charged contexts lower thresholds and speed the detection of affect [11,20,21,22]. These frameworks motivate four hypotheses. (H1) If semantically meaningful contexts generally enhance engagement with social stimuli, both disaster and positive-scene adaptation should facilitate classification of emotional over neutral faces relative to scrambled controls, consistent with sensitization. (H2) If threat-related contexts preferentially sharpen processing of threat-signaling expressions, disaster adaptation should produce a larger facilitation for fear and anger than for happiness. (H3) If real-world disaster exposure fosters “emotional expertise” and vigilance, previously exposed individuals should show a global advantage in emotion detection. Finally, (H4) if pre-traumatic stress (Pre-Cl) indexes anticipatory vigilance, higher scores should predict larger adaptation-related gains, particularly in threat-congruent conditions. By combining a tightly controlled adaptation paradigm with measures of real-world exposure and predispositions, the study aims to clarify how short-term contextual tuning and longer-term experiential factors jointly shape the detection of facial emotion—a fundamental communicative behavior in high-threat situations.

## 2. Materials and Methods

### 2.1. Participants

Fifty healthy volunteers took part in the study (mean ± standard deviation of age: 20.78 ± 4.33 years; 25 females). All participants were students, reported no psychiatric or neurological disorders, and had normal or corrected-to-normal vision. After providing informed consent, they completed a demographic survey and indicated whether they had ever personally experienced at least one natural disaster in their real-world life (e.g., flood, earthquake, avalanche, tsunami, landslide, inundation, lightning storm, fire, hurricane, volcanic eruption; additional events could be added). Eighteen participants (12 females) reported no personal experience of natural disasters, whereas 32 participants (13 females) reported having lived at least one natural disaster first-hand. Participants then completed the Pre-Cl [25], which indexes stress reactions to potentially dangerous events on a 0–4 scale (total score 0–80, higher scores indicating greater pre-traumatic stress). Pre-Cl scores ranged from 1 to 62. For analytical purposes, we split the sample at a score of 31, yielding a Low Pre-Cl subgroup (N = 27; M = 16.50, SD = 8.47; 15 females) and a High Pre-Cl subgroup (N = 23; M = 44.23, SD = 9.82; 10 females). This 2 × 2 grouping (Events: yes/no; Pre-Cl: low/high) resulted in four subgroups that differed in both disaster exposure and anticipatory stress.

### 2.2. Stimuli

**Figure 1:**
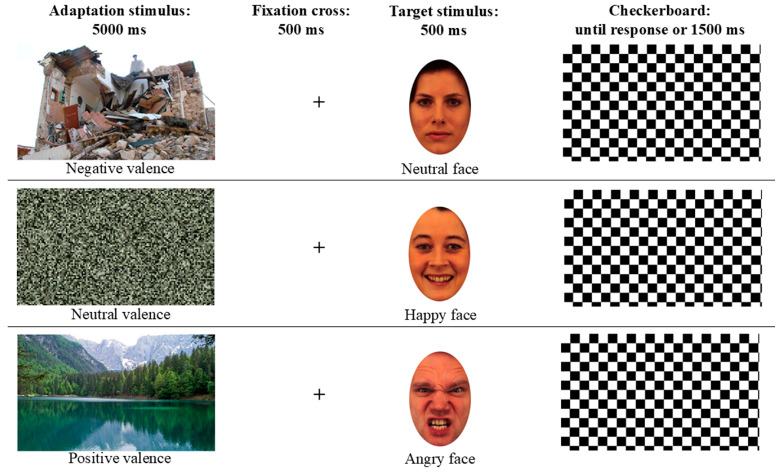
Example of adaptation trials: an adaptation stimulus was shown for 5 s with either Negative (**upper row**), Neutral (scrambled image, **central row**), or Positive (**lower row**) valence, and then a fixation cross was presented (500 ms), followed by the target face shown for 500 ms with either a neutral (**upper row**) or emotional pose (happy, angry, or fearful). Participants’ responses were recorded during the final time window of 1500 ms (checkerboard).

Adaptation stimuli were selected from the Environmental Risks to Humans (EaRTH) database [26]. From the 200 photographs of natural scenes contained in the database, 24 images were selected for the present study based on their emotional valence ratings on a 9-point Likert scale in the original study (from very negative to very positive). We selected 12 stimuli depicting natural disasters (6 images of earthquakes and 6 images of floods; mean valence score: 3.31) and 12 stimuli depicting relaxing scenes (mean valence score: 8.05). Valence scores significantly differed between the two categories (t(22) = 14.3, *p* < 0.001). Furthermore, 6 negative and 6 positive stimuli were used to create 12 scrambled images, in which the content was not recognizable. Thus, the adaptation set comprised 36 images: 12 negative, 12 positive, and 12 neutral (scrambled) stimuli. Stimuli were presented in color and measured 960 × 720 pixels (see Figure 1).

Target stimuli were extracted from the Karolinska Directed Emotional Faces (KDEF) database [27], which contains photographs of female and male actors in neutral and emotional poses. In the present study, photographs in frontal view of four female and four male actors were presented in neutral pose and with angry, fearful, and happy expressions, for a total set of 32 facial stimuli (eight actors in four poses). Faces were presented in color and were inserted into a white oval-shaped frame to hide hairstyles, leaving the face visible in an area of 300 × 460 pixels (see Figure 1).

### 2.3. Procedure

The task was administered using E-Prime 3.0 software (Psychology Software Tools Inc., Pittsburgh, PA, USA) on a Windows laptop with a screen resolution of 1280 × 768 pixels. Participants completed the task individually in a quiet room. They were asked to read and sign the informed consent form, to complete the survey and the Pre-Cl, and were then administered the computerized adaptation paradigm.

In the survey, after demographic information, participants were asked to report whether they had experienced a natural disaster in their real-world life, including flood, earthquake, avalanche, tsunami, landslide, inundation, lightning storm, fire, hurricane, or volcanic eruption (they could also add other disasters not present in the list). Then they completed the Pre-Cl. The Pre-Cl is a 20-item questionnaire assessing psychological reactions to potentially dangerous events. Previous studies have shown that its scores are significantly correlated with measures of PTSD [25]; it can therefore be considered a potential tool to predict stress-related reactions in populations exposed to natural disasters (without a PTSD diagnosis) [28]. The questionnaire investigates respondents’ feelings over the last month, using a 0–4 Likert scale, with a total score ranging from 0 to 80 (higher scores correspond to higher pre-traumatic reactions, i.e., intrusive involuntary images of possible future stressful events). The authors who developed the questionnaire define pre-traumatic stress reactions as disturbing future-oriented cognitions and images that can be part of PTSD, assessed by reversing the temporal focus of the past-directed items typically used in PTSD diagnosis. Pre-Cl was used here to quantify stress reactions to potentially dangerous events both in individuals who had personally experienced natural disasters and in those who had not. This procedure yielded four subgroups: low vs. high pre-traumatic stress, crossed with presence vs. absence of disaster exposure. This design allowed us to disentangle effects of real-world disaster experience from those of individual proneness to trauma-related stress. In addition, we exploited an adaptation paradigm to investigate the effect of momentary perceptual exposure to images of disasters (or neutral and positive scenes) on emotional face recognition.

Each trial began with the presentation of the adaptation stimulus in the center of the screen for 5 s. The adaptor could be negative (natural disaster), neutral (scrambled image), or positive (relaxing scene). After the adaptor, a black fixation cross was presented in the center of a white screen for 500 ms, followed by the target stimulus, consisting of a face that could depict either a neutral or an emotional expression, presented for 500 ms. After the target disappeared, a black-and-white checkerboard was presented until the participant responded or for a maximum duration of 1500 ms (see Figure 1). The next trial then started. Participants were instructed to maintain their gaze on the screen for the entire task and to respond to the facial stimuli by pressing either the “J” key with the right index finger or the “K” key with the right middle finger to categorize the face as neutral or emotional, respectively. They were asked to respond as quickly and accurately as possible. Participants were also informed that, at the end of the categorization task, a recognition task would be administered to verify whether they had paid attention to the adaptor stimuli (the recognition task served to ensure that participants focused on the adaptation stimuli and avoided distractions). The choice of a 5 s adaptor duration was based on prior adaptation studies showing that even brief exposures to emotional faces or scenes (often ≤5 s per trial) can induce robust, high-level aftereffects that transfer across stimuli [17,22,23,24]. Phase-scrambled versions of the same images were adopted as neutral baselines because they preserve global spatial frequency and luminance properties while removing semantic content [23,24]. Nevertheless, we acknowledge that scrambled images may differ from natural scenes in perceived structure and visual comfort, so contrasts between emotional and scrambled adaptors likely reflect a mixture of valence-related and broader contextual differences (see Conclusions).

The adaptation task comprised 288 trials: each of the 12 adaptation stimuli per category (positive, neutral, negative) was paired half of the time with a specific neutral target face and half of the time with the same identity expressing an emotional pose (two happy, two angry, and two fearful images). The association between the 12 adaptation stimuli and the target faces was repeated for negative, neutral, and positive adaptors (36 trials), and the full set was repeated for each of the eight facial identities (288 trials). In 144 trials a neutral face was the target stimulus; in the remaining 144 trials an emotional face was the target stimulus (48 happy, 48 fearful, 48 angry targets).

The order of the stimuli was fully randomized within and across participants, and overall accuracy was high, suggesting sustained engagement. However, we did not explicitly model time-on-task effects, and subtle fatigue or habituation cannot be ruled out (see Conclusions). Before the beginning of the adaptation task, four practice trials were presented to allow participants to familiarize themselves with the paradigm. After completion of the adaptation paradigm, the recognition task was administered to verify whether each participant had focused on the adaptation stimuli: 12 images were presented sequentially, and participants were instructed to press the “J” key if the stimulus had been presented as an adaptation stimulus (no response was required if the image had not been presented before). In this phase, six stimuli were new (no response required) and six had been presented during adaptation (two relaxing scenes, two earthquake images, and two flood images). After the end of the task, participants were debriefed.

The whole procedure lasted approximately 40 min. It was carried out in accordance with the principles of the Declaration of Helsinki and was approved by the Institutional Review Board of Psychology of the Department of Psychological, Health and Territorial Sciences, University “G. d’Annunzio” of Chieti-Pescara (protocol number: IRBP/20024).

### 2.4. Statistical Analysis

As a first step, accuracy in the recognition task was computed for each participant as the proportion of correct responses. Mean accuracy was 0.92 (standard deviation: 0.10). Two female participants with low Pre-Cl scores who had not experienced real-world disasters were excluded from further analysis because their accuracy in the recognition task was more than two standard deviations below the sample mean, suggesting that they did not pay sufficient attention to the adaptation stimuli (they correctly recognized 50% and 67% of the stimuli, respectively). The main analyses were therefore carried out on 48 participants’ performance in the adaptation task.

Accuracy in each condition was computed as the proportion of correct responses. Response times (RTs) were considered only for correct responses. No correct responses longer than 1000 ms were collected, and only 12 correct responses were faster than 150 ms; these 12 responses were discarded, and analyses were performed on RTs between 150 and 1000 ms. Across conditions, kurtosis values ranged from −0.31 to +0.80, indicating approximately normal RT distributions. For each condition, mean RT was divided by the proportion of correct responses to obtain the Inverse Efficiency Score (IES), with lower scores corresponding to better performance in terms of both accuracy and speed. All data are reported in the Supplementary Materials, including accuracy, RTs, and IES values.

IES was used as the dependent variable in two Analyses of Variance (ANOVAs). In the first ANOVA, Pre-Cl (low, high) and Events lived (no event, event) were used as between-subjects factors, and Adaptation (negative, neutral, positive) and Target face (neutral, emotional) were used as within-subjects factors. In the second ANOVA, Pre-Cl and Events lived were used as between-subjects factors, and Adaptation and Target emotion (happy, angry, fearful) were used as within-subjects factors; neutral targets were excluded from this analysis, with the aim of investigating the specific effect of adaptation on facial emotion processing. All statistical analyses were carried out using Statistica v. 8 software (StatSoft. Inc., Tulsa, OK, USA) and, when needed, Duncan’s test was used for post hoc comparisons, with a significant threshold set at *p* < 0.05.

## 3. Results

The first ANOVA revealed a significant main effect of Adaptation (F_(2,88)_ = 5.24, *p* = 0.008, η_p_^2^ = 0.1) and post hoc comparisons showed a better performance (lower IESs) for positive (698.5 ± 14.74) and for negative (701.36 ± 14.62) adaptation stimuli compared to neutral stimuli (715.46 ± 17.26; *p* = 0.014 and *p* = 0.033, respectively). The better performance for emotional compared to neutral Target faces did not reach significance (F_(1,44)_ = 2.91, *p* = 0.09), as well as the other main effects and interactions.

**Figure 2:**
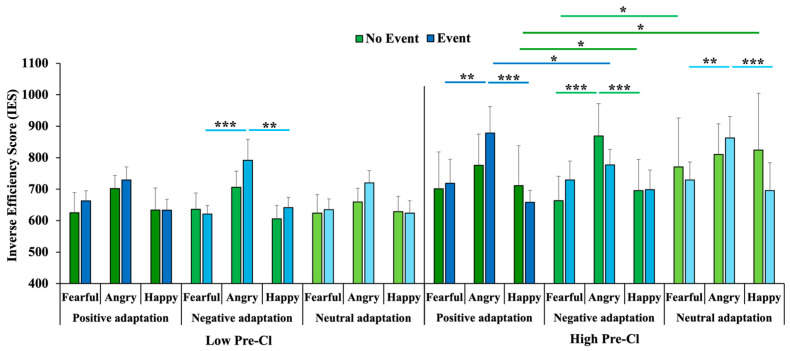
Interaction among Pre-Cl (Low, High), Events lived (No Event, Event), Adaptation (Positive, Negative, Neutral), and Target emotion (Fearful, Angry, Happy) on Inverse Efficiency Scores. Sample sizes per subgroup: Low Pre-Cl–Event, N = 15; Low Pre-Cl–No Event, N = 10; High Pre-Cl–Event, N = 17; High Pre-Cl–No Event, N = 6. Error bars represent standard errors, and asterisks indicate significant pairwise comparisons (* *p* < 0.05, ** *p* < 0.01, *** *p* < 0.001).

The second ANOVA showed a better performance for participants with low than with high pre-traumatic stress level, as revealed by the main effect of Pre-Cl (F_(1,43)_ = 4.74, *p* = 0.035, η_p_^2^ = 0.1; Low Pre-Cl: 659.94 ± 45.58; High Pre-Cl: 752.5 ± 90.74). The main effect of Target emotion was significant (F_(2,88)_ = 8.41, *p* < 0.001, η_p_^2^ = 0.16), revealing higher IESs for angry (772.72 ± 65.23) than for happy (670.17 ± 71.83) and fearful (675.77 ± 67.43) target faces (*p* < 0.001 for both comparisons). Pre-Cl significantly interacted with Adaptation (F_(2,86)_ = 3.13, *p* = 0.049, η_p_^2^ = 0.07), and post hoc comparisons showed that within the subgroup with high Pre-Cl scores, the performance was better (lower IES) when adaptation stimuli were negative than neutral (*p* = 0.04), and showed that for neutral adaptation stimuli, the performance was better for the subgroup with low Pre-Cl compared to that with high Pre-Cl scores (*p* = 0.02). Importantly, there was a significant interaction among Pre-Cl, Events lived, Adaptation and Target emotion (F_(4,172)_ = 3.47, *p* = 0.009, η_p_^2^ = 0.07; see Figure 2).

Post hoc comparisons showed that fearful and happy target faces were categorized better than angry target faces (i) when the adaptation stimulus was either positive or neutral in the subgroup with high Pre-Cl scores and personal exposure to disasters (fearful: *p* = 0.002 and *p* = 0.007; happy: *p* < 0.001 for both comparisons); (ii) when the adaptation stimulus was negative in the subgroup with high Pre-Cl but without exposure to disasters (*p* < 0.001 for both comparisons); and (iii) when the adaptation stimulus was negative in the subgroup with low Pre-Cl who had experienced disasters (*p* < 0.001 and *p* = 0.003, respectively). Moreover, only participants with high Pre-Cl scores who had experienced disasters showed better performance for angry target faces after adaptation to negative than positive stimuli (*p* = 0.041), whereas participants without personal exposure to disasters showed better performance for happy target faces after positive (*p* = 0.027) and negative (*p* = 0.012) adaptation compared with neutral adaptation, and for fearful target faces after negative compared with neutral adaptation (*p* = 0.042).

Across analyses, partial eta-squared values suggested small-to-medium effects (η_p_^2^ ≈ 0.07–0.16). Robust main effects emerged for Adaptation (η_p_^2^ = 0.10) and Target emotion (η_p_^2^ = 0.16), whereas the four-way interaction among Pre-Cl, Events, Adaptation, and Target emotion showed a smaller effect size (η_p_^2^ = 0.07). Given the sample size and subgroup structure, complex higher-order interactions should therefore be interpreted as exploratory and in need of replication, rather than as definitive evidence for experience-dependent “emotional expertise”.

## 4. Discussion

The present study investigated how short-term perceptual adaptation to emotionally salient scenes interacts with real-world exposure to natural disasters and pre-traumatic stress reactions (Pre-Cl) in shaping rapid categorization of facial expressions. In line with accounts of context-sensitive face processing and emotional vigilance, we found that both negative (disaster) and positive (relaxing) adaptors facilitated performance relative to neutral, scrambled images, as indexed by lower inverse efficiency scores (IES). This general adaptation benefit emerged across neutral and emotional targets, whereas finer-grained analyses restricted to emotional faces revealed a more complex pattern involving Pre-Cl, previous disaster exposure, and specific emotional categories. Taken together, the results provide clear support for H1, in that both disaster and positive adaptation facilitated performance relative to scrambled adaptors. H3 was not supported at the global level, as we did not observe a main effect of real-world disaster exposure on facial-emotion categorization. H2 and H4 received only partial support: threat-related facilitation for anger and fear emerged only in specific subgroups defined by Pre-Cl and exposure status, and these effects were captured by higher-order interactions of modest size. We therefore treat these patterns as preliminary and emphasize the need for replication in larger samples.

Consistent with H1, emotional adaptation (both negative and positive) improved performance relative to neutral scrambled stimuli, suggesting that exposure to semantically meaningful, affectively charged scenes transiently enhances engagement with subsequent social stimuli. This facilitatory effect did not depend on whether the target face was neutral or emotional in the first ANOVA, indicating that adaptation may have acted at a relatively general level (e.g., by increasing arousal or sharpening attentional focus on faces) rather than selectively boosting emotional over neutral expressions at the global level. This pattern is broadly compatible with behavioral work on face adaptation showing that sustained exposure to emotional material can recalibrate the perception of subsequent faces [29,30,31], and with neurocognitive models in which a distributed occipito-temporal system for face perception interacts with regions coding affective salience and context [32,33,34,35]. However, we did not collect neural or physiological data in the present study, so any reference to underlying brain mechanisms should be regarded as speculative and as a possible framework rather than a direct conclusion from our findings.

With respect to H2, the results provide a mixed picture. Across participants, angry faces yielded higher IES (i.e., worse performance) than happy and fearful faces, indicating that anger was generally more difficult to categorize as “emotional” in our binary decision task. This is somewhat unexpected, given that anger is typically considered a canonical threat cue and is often recognized efficiently, particularly when gaze and body posture are congruent [5,36]. One likely contributor is the specific decision requirement: participants judged whether a face was emotional or neutral, rather than identifying discrete emotions. In this context, certain angry identities in the KDEF set may appear closer to neutrality than fearful or happy faces, rendering anger more ambiguous. The overall disadvantage for anger may therefore reflect both stimulus-specific factors and the use of a detection rather than a labeling task. Within this general difficulty, threat-related adaptation effects did emerge in particular subgroups. Participants with high Pre-Cl who had personally experienced disasters showed better performance for angry faces after negative than positive adaptation, consistent with a selective benefit for threat-congruent cues when confronted with disaster scenes. Moreover, in participants without disaster exposure but with high Pre-Cl scores, happy faces benefited from emotional (positive or negative) relative to neutral adaptation, and fearful faces showed improved performance after negative versus neutral adaptation. These patterns suggest that negative context can selectively facilitate processing of fear (in non-exposed but high Pre-Cl individuals) and anger (in high Pre-Cl individuals with exposure), partially in line with H2. However, the absence of a robust, global advantage for threat expressions following disaster adaptation indicates that any threat-specific facilitation depends on individual differences and should be interpreted cautiously.

The findings related to Pre-Cl offer a nuanced perspective on H4. At the global level, participants with low Pre-Cl scores outperformed those with high Pre-Cl across emotional faces, as reflected by a significant main effect with lower IES in the low Pre-Cl group. This pattern suggests that heightened pre-traumatic stress reactions—characterized by intrusive future-oriented imagery and increased arousal [25]—may impose a general cognitive cost on rapid emotion categorization, possibly through greater internal distraction or less efficient allocation of attentional resources. In this sense, higher anticipatory stress does not straightforwardly translate into better “emotional expertise”; rather, it appears to reduce baseline efficiency despite increased vigilance.

At the same time, the interaction between Pre-Cl and adaptation indicates that high Pre-Cl individuals were particularly sensitive to emotional context, showing better performance after emotional than neutral adaptation. When considered together with the four-way interaction, this pattern partially supports H4 by suggesting that elevated pre-traumatic stress is associated with greater context-dependent tuning, especially when the adaptor matches the threatening content of anticipated events (disasters) and the target expression signals interpersonal threat (anger). These observations resonate with models proposing that hypervigilant individuals show exaggerated responsiveness to threat cues but reduced efficiency under neutral or ambiguous conditions, due to sustained arousal and difficulty disengaging from threat-related material [37,38,39]. Once again, however, these conclusions are based on interaction effects of modest size and should be regarded as tentative.

Regarding real-world disaster exposure (H3), we did not find evidence for a simple main effect indicating that previously exposed individuals uniformly outperform non-exposed ones in facial-emotion categorization. This diverges from some previous work reporting enhanced accuracy and faster responses across several emotions in earthquake survivors [12,13]. In our data, exposure effects emerged only in the context of higher-order interactions that also involved Pre-Cl, adaptation condition, and target emotion. For example, in high Pre-Cl individuals with disaster exposure, anger recognition benefited specifically from negative relative to positive adaptation, whereas in participants without exposure, happy and fearful faces showed robust gains after emotional adaptors compared to neutral ones. These findings suggest that real-world exposure does not produce a uniform advantage but rather modulates how trait predispositions (Pre-Cl) and immediate context (adaptation) interact to shape performance. Neuroimaging studies in trauma- and disaster-exposed samples have documented alterations in networks involved in emotion, salience, and self-referential processing [16,38,39]. Our behavioral results are consistent with the idea that pre-traumatic stress and disaster exposure may influence how contextual information is weighted during face processing, but we cannot specify the underlying neural mechanisms on the basis of the present data, and we therefore treat such links as hypotheses for future investigation rather than as established mechanisms.

More broadly, the present findings underscore that facial emotion categorization is not solely determined by static facial information but is dynamically shaped by recent contextual input and by individual histories of environmental threat and anticipatory stress. Brief exposure to emotionally salient environments influenced the speed and efficiency of subsequent emotion detection, and this influence was modulated by both pre-traumatic stress and disaster exposure. People are continuously embedded in affective contexts—physical settings, media, interpersonal climate—that precede or accompany social interactions; our data provide behavioral evidence that such contexts can bias how quickly and efficiently emotional faces are detected [4,40,41]. A reassuring, positive context may facilitate the reading of happy expressions and reduce the tendency to misinterpret ambiguous faces as negative, whereas a threat-laden context may sharpen detection of fear and anger. These dynamics are likely to interact with individual history in shaping interpersonal trust and vigilance. At the same time, given the modest effect sizes, limited sample, and reliance on a binary detection task, any applied or clinical implications remain highly speculative. We therefore see our study primarily as a contribution to basic research on context-sensitive emotion perception, with potential translational relevance to be examined in future work rather than claimed at this stage.

## 5. Conclusions

The present study examined how short-term perceptual adaptation to emotionally salient scenes combines with real-world disaster exposure and pre-traumatic stress reactions (Pre-Cl) to influence rapid categorization of facial expressions as emotional or neutral. Relative to phase-scrambled controls, both disaster-related and positive environmental scenes facilitated performance, indicating that semantically meaningful affective contexts can transiently enhance emotion detection. At the same time, complex interactions involving Pre-Cl, disaster exposure, and target emotion suggested that these benefits are not uniform: they depend on the match between adaptor and target content and on individual predispositions. Overall, the data provide clear support for a general adaptation benefit (H1) and only partial, preliminary support for threat-specific and trait-dependent hypotheses (H2–H4).

Several limitations temper the generalizability of these findings. First, the sample size and resulting subgroup cell sizes limit statistical power, especially for higher-order interactions; the four-way interaction should therefore be viewed as exploratory and in need of replication in larger and more diverse samples. Second, the adaptation paradigm contrasted disaster, positive, and scrambled scenes, but the latter—although standard in the literature—may differ from natural scenes in visual structure and subjective experience beyond emotional valence. Third, the binary emotional/neutral decision emphasizes detection rather than identification of specific emotions; this likely contributed to the difficulty with anger and constrains the scope of our conclusions [40,41]. Fourth, although accuracy was high overall, the relatively long task (288 trials) may have introduced fatigue or habituation effects that we did not explicitly model. Finally, we relied on a composite IES index; future work could complement this approach with hierarchical modeling or diffusion-model analyses that more directly capture speed–accuracy trade-offs.

Looking forward, combining similar cross-category adaptation paradigms with neurophysiological methods could clarify when and how contextual tuning influences face processing. EEG studies using advanced spatio-temporal fusion approaches [42] and cross-subject domain-adaptation techniques [43] are particularly well suited to track dynamic changes in emotion-related brain activity and to test whether adaptation effects generalize across individuals. Longitudinal and clinical studies, including trauma-exposed populations and individuals with elevated pre-traumatic stress, could examine whether the patterns observed here reflect transient states, stable traits, or modifiable risk markers. Ultimately, a better understanding of how contextual information, disaster exposure, and anticipatory stress jointly modulate facial-emotion detection may contribute to more precise models of adaptive and maladaptive responding under threat.

## Data Availability

The raw data supporting the conclusions of this article will be made available by the authors on request.

## Supplementary Materials

The following supporting information can be downloaded at: https://www.mdpi.com/article/10.3390/brainsci16010005/s1, Table S1: Raw data of the experiment.

## Abbreviations

The following abbreviations are used in this manuscript:

Pre-Cl	Pre-Traumatic Stress Reactions Checklist
PTSD	Post-Traumatic Stress Disorder
STS	Superior Temporal Sulcus
